# The Role of Dopaminergic Genes in Probabilistic Reinforcement Learning in Schizophrenia Spectrum Disorders

**DOI:** 10.3390/brainsci12010007

**Published:** 2021-12-22

**Authors:** Dorota Frydecka, Błażej Misiak, Patryk Piotrowski, Tomasz Bielawski, Edyta Pawlak, Ewa Kłosińska, Maja Krefft, Kamila Al Noaimy, Joanna Rymaszewska, Ahmed A. Moustafa, Jarosław Drapała

**Affiliations:** 1 Department of Psychiatry, Wroclaw Medical University, Pasteur Street 10, 50-367 Wroclaw, Poland; tomaszbielawski90@gmail.com (T.B.); maja.krefft@gmail.com (M.K.); kamila.kotowicz@gmail.com (K.A.N.); joanna.rymaszewska@umw.edu.pl (J.R.); 2 Department of Psychiatry, Division of Consultation Psychiatry and Neuroscience, Wroclaw Medical University, Pasteur Street 10, 50-367 Wroclaw, Poland; mblazej@interia.eu (B.M.); tryku@yahoo.com (P.P.); 3 Department of Experimental Therapy, Institute of Immunology and Experimental Therapy, Polish Academy of Sciences, Weigel Street 12, 53-114 Wroclaw, Poland; edyta.pawlak@hirszfeld.pl; 4 Day-Care Psychiatric Unit, University Clinical Hospital, Pasteur Street 10, 50-367 Wroclaw, Poland; ewa.klosinska@gmail.com; 5 School of Psychology, Marcs Institute for Brain and Behaviour, Western Sydney University, Locked Bag 1797, Penrith, NSW 2751, Australia; ahmedhalimo@gmail.com; 6 Department of Human Anatomy and Physiology, The Faculty of Health Sciences, University of Johannesburg, Johannesburg 2006, South Africa; 7 Department of Computer Science and Systems Engineering, Faculty of Information and Communication Technology, Wroclaw University of Science and Technology, Wybrzeze Wyspianskiego Street 27, 50-370 Wrocław, Poland; jaroslaw.drapala@gmail.com

**Keywords:** schizophrenia, reinforcement learning, probabilistic learning task, striatum, basal ganglia, prefrontal cortex

## Abstract

Schizophrenia spectrum disorders (SZ) are characterized by impairments in probabilistic reinforcement learning (RL), which is associated with dopaminergic circuitry encompassing the prefrontal cortex and basal ganglia. However, there are no studies examining dopaminergic genes with respect to probabilistic RL in SZ. Thus, the aim of our study was to examine the impact of dopaminergic genes on performance assessed by the Probabilistic Selection Task (PST) in patients with SZ in comparison to healthy control (HC) subjects. In our study, we included 138 SZ patients and 188 HC participants. Genetic analysis was performed with respect to the following genetic polymorphisms: rs4680 in *COMT*, rs907094 in *DARP-32*, rs2734839, rs936461, rs1800497, and rs6277 in *DRD2*, rs747302 and rs1800955 in *DRD4* and rs28363170 and rs2975226 in *DAT1* genes. The probabilistic RL task was completed by 59 SZ patients and 95 HC subjects. SZ patients performed significantly worse in acquiring reinforcement contingencies during the task in comparison to HCs. We found no significant association between genetic polymorphisms and RL among SZ patients; however, among HC participants with respect to the *DAT1* rs28363170 polymorphism, individuals with 10-allele repeat genotypes performed better in comparison to 9-allele repeat carriers. The present study indicates the relevance of the *DAT1* rs28363170 polymorphism in RL in HC participants.

## 1. Introduction

Schizophrenia (SZ) is a complex disorder with diverse symptomatology, including positive symptoms, negative symptoms, and cognitive dysfunction [1,2,3]. Most research on schizophrenia has focused on dopaminergic circuitry abnormalities as a key mechanism underlying the pathogenesis of disorder [4]. Dopamine (DA) has been linked to both positive and negative SZ symptoms [5], with irregular DA release hypothesized to ascribe aberrant salience to irrelevant stimuli [6]. Disrupted DA function has been hypothesized to prevent appropriate reinforcement learning (RL) [4]. RL relies on modifying expectations and behavior following positive and negative outcomes so that adaptive actions are more likely to be repeated in the future, whereas maladaptive actions are more likely to be suppressed over time [7]. Positive outcomes reflected in terms of deviations from current expectations are called positive prediction error (PE), which is encoded by phasic bursts of DA [8]. Similarly, negative PE are encoded by phasic dips or pauses in dopaminergic activity when rewards are expected but not received [9]. These phasic bursts and dips in DA modify synaptic plasticity in the connections between prefrontal cortical areas (PFC) and the basal ganglia (BG) (mainly striatum), allowing the system to incrementally become more likely to select actions that are adaptive and avoid actions that are maladaptive [10].

Psychotic symptoms in SZ could be partially understood in terms of faulty PE signals that fail to discriminate between adaptive and nonadaptive associations, giving attention to irrelevant stimuli that in fact should be ignored [11]. This is manifested by poor performance on latent inhibition, blocking, overshadowing, and learned irrelevance tasks in patients with SZ (for a review see [12]). Moreover, functional imaging studies have shown that striatal reinforcement PE signals are disrupted in patients suffering from psychotic symptoms [13,14]. In the probabilistic RL paradigms, patients with SZ have been shown to have relatively impaired learning from positive PE with spared learning from negative PE [15]. Moreover, it has been shown that patients with SZ fail to show striatal DA-dependent implicit tendency to speed response in the face of high-reward incentives [16]. It has been reported that patients with SZ and a high level of negative symptoms compared to patients with SZ and a low level of negative symptoms have difficulty in using positive expected values to guide novel choices [17]. Results from a recent study on both medicated and unmedicated patients with SZ suggest that both groups of SZ patients show overreliance on PE-driven learning and have less dependence on learning explicit value representations, while the unmedicated group show additionally greater decision noise in comparison to healthy control participants [18].

Genes related to dopaminergic neurotransmission are associated with the risk of developing SZ, and their relevance to the development of SZ have been confirmed in the genome-wide association studies (GWAS) [19]. The dopaminergic D2 receptor gene and those that are involved in the upstream regulation of DA synthesis are among many genes associated with the risk of SZ [20]. On the other hand, RL has been suggested as a candidate for an intermediate phenotype in SZ [21]. Recent studies have demonstrated that individuals at increased clinical risk of developing psychosis are characterized by subtle PE abnormalities during RL task performance [22], and patients with first-episode psychosis (FEP) present lower learning rate as well as lower sensitivity to reward and punishment in RL tasks [23]. It was reported in a neuroimaging study that polygenic risk score for SZ (PRS) is associated with striatal activation during reward anticipation among healthy adolescents [24], but PRS was not shown to be associated with performance on RL in the general population [23]. Until now, dopaminergic genes associated with prefrontal cortical and striatal dopamine function have been shown to be predictive of individual differences in RL with respect to ability to learn from rewards and punishments as well as to adapt behavior on a trial-to-trial basis, respectively [25,26,27,28,29,30,31,32].

The most widely studied dopaminergic genes with respect to RL are genes associated with prefrontal level of DA (*COMT*, *DRD4*) and striatal dopaminergic D1 and D2 receptors functionality (*DARPP-32*, *DRD2*, *DAT1*). It has been found that the *COMT* gene which is linked to PFC DA level is also associated with trial-to-trial adjustments after negative feedback [25], and with accuracy in learning during trials with incongruent coupling of action and valence [30]. In one meta-analysis, the *COMT* gene was also confirmed to be associated with reward learning in RL tasks [31]. In turn, the *DARP-32* gene associated with D1-dependent synaptic plasticity in the striatum has been shown to predict decisions probabilistically associated with positive outcomes [25,27]. On the other hand, the *DRD2* gene, affecting postsynaptic D2 receptor density in the striatum without affecting presynaptic DA function, has been shown to influence learning to avoid decisions that are probabilistically associated with negative outcomes [25,27,29], and to predict learning to inhibit an action to obtain rewards [30].

Although there is a body of research showing the relevance of the dopaminergic PFC-BG circuitry to RL performance in the healthy population and the association of genes related to dopaminergic neurotransmission with the risk of SZ development and SZ symptomatology with reinforcement sensitivity, it has not been shown whether variation in dopaminergic genes is associated with RL in SZ. Therefore, in the present study, we aimed to examine a differential impact of variants in dopaminergic genes (rs4680 in *COMT* gene, rs747302, rs1800955 in *DRD4* gene, rs907094 in *DARP-32* gene, rs2734839, rs936461, rs1800497, rs6277 in *DRD2* gene, rs28363170, rs2975226 in *DAT1* gene probabilistic RL task performance in patients with SZ compared to healthy controls.

## 2. Materials and Methods

### 2.1. Participants

In our study, we included 138 patients with schizophrenia spectrum disorders: 18% with schizoaffective disorder, 62% with paranoid schizophrenia and 20% with first-episode psychosis (58 males/52 females, aged 37.61 ± 12.99 years) and 188 healthy controls (66 males/122 females, aged 39.07 ± 18.74 years). A diagnosis of SZ was based on the DSM-IV and ICD-10 criteria, validated using the Operational Criteria for Psychotic Illness (OPCRIT) checklist [33]. All patients were of Caucasian origin and were recruited from the hospitals and out-patient units in the Lower Silesian area, Wroclaw, Poland. The exclusion criteria were as follows: general brain disorder, intellectual disability, severe physical health impairments and comorbid drug and/or alcohol use disorder (except of nicotine dependence). The study was approved by the Wroclaw Medical University Ethics Committee and all participants gave written informed consent.

### 2.2. Measures

#### 2.2.1. Clinical Assessment

Clinical manifestation was assessed using the Brief Psychiatric Rating Scale (BPRS) (Overall and Gorman, 1962), the Scales for the Assessment of Negative Symptoms (SANS) and Positive Symptoms (SAPS) [34], the Positive and Negative Syndrome Scale (PANSS) [35], the Montgomery–Asberg Depression Rating Scale (MADRS) [36], and the Hamilton Depression Rating Scale (HDRS) [37]. General functioning was recorded using the Global Assessment of Functioning scale (GAF) (American Psychiatric Association, 1994). The dosage of antipsychotics was expressed as chlorpromazine equivalents (mg/day) [38].

#### 2.2.2. General Neuropsychological Assessment

Participants were assessed with respect to cognitive performance on the Repeatable Battery for the Assessment of Neuropsychological Status (RBANS) [39]. The RBANS is a brief, neuropsychological screening measure. It consists of 12 subtests that can be combined into five domains: immediate memory (list learning and story memory), visuospatial/constructional (figure copy and line orientation), language (picture naming and semantic fluency), attention (digit span and coding), and delayed memory (list recall, list recognition, story recall, and figure recall).

#### 2.2.3. Probabilistic Selection Task

To assess RL, we used a computerized version of the Probabilistic Selection Task (PST) [40]. The task has training and test phases. During the training phase, participants are required to choose between three stimulus pairs presented in a random order (AB, CD, EF). In each trial, a participant makes a choice between stimulus pairs. These stimulus pairs were followed by predetermined probabilistic feedback (80:20%, 70:30%, 60:40%). In the test, Japanese Hiragana characters are used as stimulus pairs to minimize explicit verbal encoding. Acquisition blocks consisted of 30 trials. The training phase was terminated when participants either achieved a criterion as defined by 65% correct in the AB (80:20) condition, 60% correct in the CD (70:30) condition, and 50% correct in the EF (60:40) condition or after 120 trials were completed. This criterion was intended to prevent overlearning of the contingencies prior to the test phase [41]. Following training phase, participants completed test phase involving 75 combinations of paired stimuli, of which 15 consisted of prior parings and 60 involved novel combinations. No feedback was provided during this phase. During the test phase, we analyzed the participants’ performance using old and novel test pairings involving an A or B stimulus (AB, AC, AD, AE, AF and BC, BD, BE, BF). Positive-reinforcement learning (Go) was assessed by the choose-A frequency, while avoidance learning (NoGo) is assessed by the avoid-B frequency in the old and novel pairs.

### 2.3. Genotyping

We analyzed functional polymorphisms, including single nucleotide polymorphisms (SNPs) and variable number of tandem repeats (VNTR) polymorphisms, that have been shown to be associated with schizophrenia and/or affect dopaminergic neurotransmission: the *COMT* gene Val158Met (rs4680), the *DARPP-32* gene (rs907094), the *DRD2* gene (rs2734839, rs936461, rs1800497 and rs6277), the *DRD4* gene (rs747302 and rs1800955), the *DAT1* gene (rs28363170 and rs2975226). The polymorphism Val158Met (rs4680) in the *COMT* gene resulting in G-to-A transition at codon 158 of the *COMT* gene causes a valine-to-methionine (Val158Met) substitution. Met allele carriers have lower COMT enzyme activity and higher prefrontal DA levels [42]. The polymorphism rs907094 in the *DARPP-32* (*PPP1R1B*) gene resulting in C-to-T transition influences the abundance of intracellular DARPP-32 mRNA and modulates striatal activity as well as function [42]. Polymorphism rs1800497 in the *DRD2* gene (*DRD2/ANKK1* Taq1A, C32806T Glu713Lys) is associated with DA receptor density and availability [43,44]. The polymorphism rs2734839 in the *DRD2* gene is associated with A-to-G transition and has been shown to be associated with risk of schizophrenia [45]. The polymorphism rs6277 in the *DRD2* gene affects DRD2 mRNA stability [46] and has been found to predict striatal DRD2 availability [47]. It influences striatal D2 postsynaptic receptor density without affecting presynaptic DA function [48]. Polymorphism rs1799732 in the *DRD2* gene (−141 Ins/Del) has been associated with striatal receptor D2 density [49] and striatum response to reward [50]. Polymorphism rs1800955 of the *DRD4* gene, resulting in C-to-T transition, has been shown to influence transcriptional efficiency by 40% [51] and is predictive of error-related prefrontal activity and compensatory behavioral adjustments following these errors [52]. Polymorphism rs747302 in the *DRD4* gene has been found to influence reduced transcription and to lower DA receptor density [53]. Polymorphism rs2975226 in the *DAT1* gene is associated with A-to-T transition and has been significantly associated with schizophrenia [54]. The polymorphism rs28363170 in the *DAT1* gene has been associated with reduced expression of dopamine transporter protein, resulting in relatively increased extrasynaptic striatal dopamine levels in the former [55,56]. It is predictive of brain and behavioral responses to cognitive flexibility [57], modulates striatal activation as a function of working memory load [58] and influences implicit learning [59].

Venous blood samples were collected from participants who agreed to genetic analysis. Genomic DNA was isolated from peripheral white blood cells using the DNA Blood Midi Kit (Qiagen). The SNPs in the following genes: *DRD2* (rs2734839, rs1800497, rs1799732, rs6277), *DRD4* (rs936461, rs1800955), *COMT* (rs4680) and *DARPP-32* (rs907094) were genotyped with the allelic discrimination (AD) technique with the use of validated and predesigned TaqMan SNP Genotyping Assays ((C___2601170_10, C___7486676_10, C__33641686_10, C__11339240_10, C___7470693_30, C___7470700_30, C__25746809_50 and C___7452370_1_, respectively) according to the manufacturer’s instructions (Thermo Fisher Scientific Inc., Waltham, MA, USA). The PCR-RFLP technique was used for the *DAT1* rs2975226 (*Psyl*) polymorphism. The *DAT1* VNTR polymorphism (rs28363170) was genotyped using PCR with a pair of primers, where the forward one was labeled with 6-FAM followed by capillary electrophoresis in the presence of the GeneScan™ 600 LIZ^®^ Size Standard on 3500 Genetic Analyzer (Thermo Fisher Scientific Inc., USA). Individual genotypes, i.e., the 9-allele (440 bp) and the 10-allele (480 bp) ones, were detected according to the peak size on the GeneMapper^®^ Software version 4.0 (Thermo Fisher Scientific Inc., Waltham, MA, USA).

### 2.4. Statistical Analysis

The comparison of general characteristics between HC and SZ patients was performed using the analysis of variance (ANOVA) in case of continuous variables and the *χ*^2^ test in case of categorical variables. The comparison of genotype and allele distribution between HC and SZ was performed using the *χ*^2^ test. Similarly, the *χ*^2^ test was used to test whether genotype distributions were in agreement with the Hardy–Weinberg equilibrium (HWE). The ANOVA was also applied to assess the association between studied genetic polymorphisms and performance during the training and test phases of the reinforcement task. Bonferroni correction was used due to multiple testing (10 SNPs), the significant *p*-value for genetic testing was below 0.004.

To compare the acquisition of contingencies between SZ patients and HC participants, a two-way ANOVA with factors of group and reinforcement probability as well as appropriate post hoc tests were performed on participants‘ performance on PST (Levene’s test of homogeneity *p*-value greater than 0.05). In order to compare overall probabilistic selection performance, we created a summary measure by averaging the proportion of correct responses from all three conditions of each stimulus pair (AB, CD, EF) and we used a *t*-test to assess difference between SZ patients and HC participants. As a test of general neuropsychological functioning and experimental task performance, we used gender, educational level and RBANS total score as covariates in analysis of covariance (ANCOVA) with the effects of group (SZ vs. HCs) and reinforcement probability as independent variables. The learning of probabilistic contingencies was also assessed at the test phase using ANOVA, with the effects of group (SCZ vs. HCs) and reinforcement probability as factors. Group differences in test phase performance were assessed using *t*-tests for the choose-A frequency (Go) and the avoid-B frequency (NoGo) generated from cumulative test phase scores on the pairs involving A (Go) and pairs involving B (NoGo).

In correlational analyses of continuous variables, we used Pearson’ and Spearman’s correlation coefficients depending on the normality of distribution of the given variable (Kolomogorov-Smirnov test *p*-value greater than 0.05). We performed correlation analyses to assess relationships between performance on PST and clinical symptom ratings (SANS, SAPS, PANSS, MADRS, BPRS, HDRS), general functioning (GAF), chlorpromazine equivalent dosage and neurocognitive functioning (RBANS). We also used correlation to assess associations between RBANS and clinical symptomatology, general functioning, and chlorpromazine equivalent. All tests were two-tailed with the level of significance was set at *p*-value less than 0.05. Statistical analysis was performed using the Statistical Package for Social Sciences, version 20 (SPSS Inc., Chicago, IL, USA).

## 3. Results

The general characteristics of the sample are presented in Table 1. The distribution of specific genotypes followed the HWE (*p*-value > 0.05), except for two polymorphisms: DRD4 rs747302 in SZ patients and DAT1 rs2975226 among HC participants, which were excluded from further analysis. There were no significant differences in genotype distributions between patients with SZ and HC participants with respect to all studied polymorphisms (*p*-value > 0.05) (Table A1). Cognitive assessment on RBANS was completed by 110 SZ patients and 188 HC participants. The RL task was completed by 59 SZ patients and 95 HC participants. We excluded eight SZ patients and five HC participants due to high numbers of omissions either in the training phase or test phase of PST.

**Figure 1:**
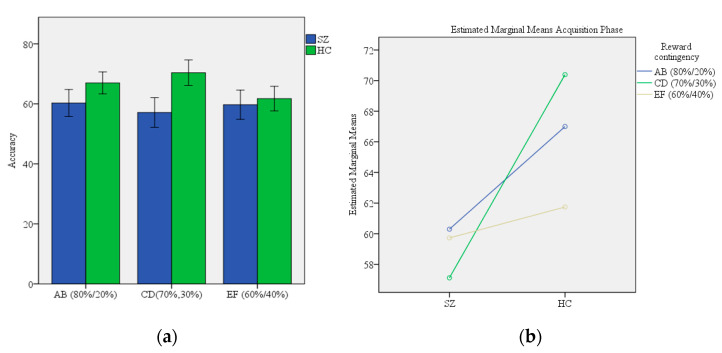
The comparison of performance on PST (**a**) Acquisition of probabilistic contingencies during training phase of PST for AB, CD and EF conditions in SZ patients and HC participants.; (**b**) Effects of group and reward contingency on acquisition accuracy of probabilistic contingencies during PST for AB, CD and EF conditions in SZ patients and HC participants. Abbreviations: PST—Probabilistic Selection Task, group: healthy control (HC), schizophrenia (SZ), reward contingency: AB (80%/20%), CD (70%, 30%), EF (80%, 20%).

A *t*-test assessing the difference between SZ patients HC participants on the summary measure created by averaging the proportion of correct responses from all three conditions of each stimulus pair (AB, CD, EF) during the training phase of PST showed better overall performance of SZ patients (59.04 ± 11.76) in comparison to HC participants (66.38 ± 12.81) (*df* = 139, *p*-value = 0.001). Two-way ANOVAs for accuracy during the training phase of PST showed a significant main effect of group (F = 15.52, *p*-value < 0.0001), no main effect of reward contingency (F = 1.13, *p*-value = 0.324), and a significant group x reward contingency interaction (F = 3.07, *p*-value = 0.047) (Model 1, Table 2). Post hoc tests revealed that HC participants performed better than SZ patients in the CD (70%/30%) condition (70.39 ± 20.33 and 57.12 ± 17.63, respectively) (*p*-value = 0.001). Proportions of correct responses given by participants during the training phase of PST with respect to each condition (AB, CD, EF) are shown in Figure 1a. Effects of group and reward contingency on acquisition accuracy of probabilistic contingencies during PST for AB, CD and EF conditions in SZ patients and HC participants are shown in Figure 1a,b.

Correlational analyses between averaged performance measure on the RL task across all reward conditions (AB, CD, EF) in the whole group showed an association with total RBANSS score (*r* = 0.30, *p* < 0.001), in particular with measures of immediate memory (*r* = 0.30, *p*-value < 0.001), attention (*r* = 0.26, *p*-value = 0.002) and delayed memory (*r* = 0.30, *p*-value < 0.001) (Table A2). Among SZ patients, we found no significant associations between performance during the training phase on PST and clinical measures (SAPS, SANS, BPRS, MADRS, BDI), general functioning (GAF), chlorpromazine equivalent dose (CPZ), or age of onset or illness duration (*p* > 0.05) (Table A2). However, we found significant negative associations between RBNAS total score and PANSS negative symptoms (*r* = −0.34, *p*-value = 0.001), PANSS general symptoms (*r* = −0.250, *p*-value = 0.049), PANSS total score (*r* = −0.33, *p*-value = 0.001), BPRS (*r* = −0.26, *p*-value = 0.021) and CPZ (*r* = −0.23, *p* = 0.027) as well as significant positive association between RBANS total score and GAF (*r* = 0.29, *p* = 0.011) (Table A3).

There were significant differences in the distribution of gender and educational level in our samples, so we included these variables as covariates in an ANCOVA analysis. We recoded education level into two categories—people with and without higher education. In the ANCOVA models, we found that the gender was not a significant covariate with respect to averaged performance on the training phase of PST, while the educational level proved to be a significant covariate (Model 2, Table 2). We found that the use of RBANS total score and educational level as covariates in an ANCOVA was a significant variable in the effects of group and reward contingency on task performance (Models 3 and 4, Table 2). However, when we used all the covarying variables (gender, educational level and RBANS total score), the model was explaining the highest variance and these covarying variables were not significant, while the interaction of the group (SZ, HC) and reward contingency (AB, CD, EF condition) were significant on the trend level (*p*-value = 0.056).

Group differences in test phase performance using t-tests for measures of choose-A frequency (Go) and avoid-B frequency (NoGo) generated from cumulative test phase scores on the pairs involving A (Go) and pairs involving B (NoGo) showed no statistically significant difference in choose-A frequency (*p*-value = 0.650) nor in avoid-B frequency (*p*-value = 0.147). Correlational analysis showed no significant associations of choose-A nor avoid-B frequencies with clinical variables (BPRS, PANSS, SAPS, SANS, RBANS, MADRS, GAF, CPZ, HDRS). Importantly, there was a significant negative association between choose-A and avoid-B frequency among SZ patients (*r* = −0.39, *p* = 0.005), while among HC participants there was no significant association between choose-A accuracy and avoid-B accuracy (*r* = −0.02, *p*-value = 0.858).

**Figure 2:**
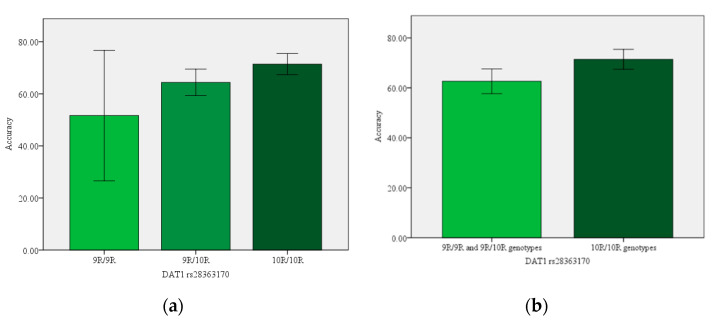
Effects of *DAT1* rs28363170 polymorphism on acquisition accuracy of probabilistic contingencies during PST for AB, CD and EF conditions in HC participants: (**a**) with respect to 9R/9R, 9R/10R and 10R/10R genotypes; (**b**) with respect to 9R allele carriers (9R/9R and 9R/10R genotypes) and 10R/10R genotypes.

The results of analysis of variance (ANOVA) showing the associations between studied genetic polymorphisms and performance during the training and test phases of the RL task are shown in Table 3. There was a significant association between *COMT* rs4680 polymorphism and averaged learning performance in the training phase of PST (*p*-value = 0.035) among HC participants. Further analysis did not show significant differences in accuracy between *COMT* rs4680 polymorphism Met allele carriers (Met/Met and Met/Val genotypes) in comparison to individuals with Val genotypes (*p*-value > 0.050). There were significant associations between the DRD4 rs1800955 polymorphism and choose-A frequency in the test phase of PST in the whole group (*p*-value = 0.039) and among HC (*p*-value = 0.047) participants; however, post hoc tests did not show any statistically significant differences between genotypes (*p*-value > 0.005). Moreover, there was a significant association between the *DAT1* rs2975226 polymorphism and averaged learning performance in the training phase of PST among SZ patients (*p*-value = 0.018) and *DAT1* rs28363170 polymorphism and averaged learning performance in the training phase of PST in the whole group (*p*-value = 0.042) and among HC participants (*p*-value = 0.004). Additional analysis did not show better accuracy in overall learning among A allele carriers and individuals with TT genotypes of the *DAT1* rs2975226 polymorphism among SZ patients (*p* > 0.05); however, there was worse accuracy in overall learning on PST among 9-allele carriers in comparison to 10-allele genotypes of the *DAT1* rs28363170 polymorphism among HC participants (*p*-value = 0.007) (Figure 2). However, it was not significant after applying Bonferroni correction (*p*-value > 0.004).

## 4. Discussion

Results from the current study are consistent with earlier research, showing an overall impairment of probabilistic RL in patients with SZ compared to HC participants [60,61,62,63,64,65]. For a long time now, the inability of patients with schizophrenia to adopt to environmental conditions flexibly and adequately has been associated with deficits in feedback-driven learning and reinforcement-based decision making [17,66]. The PST using Hiragana characters used in order to reduce verbal encoding of stimuli originally used in patients with Parkinson’s disease [40] has been used a few times in SZ samples, showing their impaired performance compared to HC participants [41,67,68]. Moreover, the use of images of common objects produced similar results in the PST task [18,41,69].

Research on learning from rewards or punishments and impairment severity have yielded mixed results [70]. Most previous research has found that RL deficits are mainly due to impairment in learning from positive feedback [41,66,69,71]; however, we observed a similar level of performance in both positive-reinforcement learning (Go) and avoidance learning (NoGo) in the test phase of PST among patients with SZ, which is in agreement with some previously reported results [67,68]. However, in our study, we observed worse performance of patients with SZ in comparison with HC only during the CD (70%/30%) condition, which may explain our results of similar performances in the testing phase, which included either A or B stimuli that were used to assess Go and NoGo tendency in PST. Based on computational modeling studies, it has been suggested that learning impairments from positive PE can be accounted for by reduced striatal D1 receptor function, compounded by noisy phasic DA signals that do not appropriately signal the positive PE magnitude [10]. On the other hand, relatively spared learning from negative PE in SZ has been attributed to the striatal D2 receptor blockage by antipsychotics [72]. However, antipsychotic drugs vary widely in their affinity to different receptor types, with second-generation antipsychotics having weaker affinity to D2 receptors but greater affinity to D1 receptors in comparison with first-generation antipsychotics [73]. Specifically, D1 receptor occupancy by clozapine is thought to contribute to its atypical properties [74]. Moreover, it has been shown that different types of antipsychotic drugs exert different impairments depending on cognitive domain [75]. Additionally, antipsychotic drugs, including clozapine, have been shown to be effective in treating cognitive dysfunctions through genetic-driven dopaminergic mechanisms [76].

Contrary to our expectations, we did not find significant correlations between RL and negative symptoms which have previously been identified in patients with SZ [17,41,71]. Inconsistencies among findings may reflect differences in sampling, since prior studies recruited patients with SZ and predominantly negative symptoms [41], or a smaller percentage of patients with schizoaffective disorder [17,77]. Moreover, it has been argued that RL processing is associated more with primary than secondary causes of negative symptoms (e.g., depression, anxiety, disorganization), and thus the duration of illness might be a reason for obtaining conflicting results showing the association of negative symptoms with RL among chronic SZ patients, but not FEP samples [78]. Therefore, the inclusion of patients with FEP might have attenuated the association between negative symptoms and learning performance.

There are several behavioral and neuroimaging studies linking candidate genes involved in the etiology of SZ with RL performance in HC participants [25,30,79,80]. In our study, we have shown that the *DAT1/SLC6A3* rs28363170 polymorphism is associated with RL performance on PST among HC participants. This is in line with research showing that a relatively large proportion of the variance in higher cognitive functions across the population can be accounted for by genetic factors [81]. The *DAT1* gene contains a variable number of tandem repeats (VNTR) polymorphism, with the two most frequent alleles in the population being nine- and ten-repeat (9R and 10R) alleles [82]. Several in vitro studies suggest that the 9R allele relative to the 10R allele of the *DAT1* rs28363170 polymorphism is associated with a reduced expression of DA transporter protein (DAT) [55,56,83], while in vivo, single-photon emission computed topography (SPECT) studies have produced mixed results, with a recent meta-analytic study showing that the 9R allele is associated with increased DAT expression, and thus potentially more efficient reuptake of DA in comparison with the 10R variant [84]. DAT is primarily expressed in the striatum, with only scarce expression in the prefrontal cortical areas [85]. Lower density of DAT in the striatum results in relatively increased extrasynaptic striatal DA availability among carriers of the 9R allele compared to 10R/10R genotypes [86,87,88]; however, opposite results have also been reported [89].

The *DAT1* VNTR functional polymorphism, which has been shown to be predictive of neural and behavioral responses to cognitive flexibility [57], modulates striatal activation as a function of working memory load [58], influences implicit learning [59] and extinction of conditioned fear responses [90]. Neuroimaging studies demonstrated that DAT availability in the striatum is correlated with neural response to reward anticipation in the nucleus accumbens [91], and the *DAT1* rs28363170 polymorphism has been associated with reward-related activity, reported to be associated with the 9R allele [50,92,93,94]. It has been shown that the *DAT1* rs28363170 polymorphism is associated with PE-based learning [88,89,90,93], but also with instructional control [32], task-switching [93] and perseveration after reversal of reinforcement contingencies [95]. Discrepancies in findings could be associated with the fact that it is not clear how genetic variation in the *DAT1* gene influences tonic and phasic DA levels [32]. Phasic DA changes are related to salient stimuli and have been shown to enable RL based on PE [8,9], while tonic DA release has been associated with exploitation of RL [96] or weighing of effort costs [97]. Moreover, there are reciprocal relationships between tonic DA levels and phasic DA burst in striatal areas [98]. Future neuroimaging studies should attempt to disentangle the genetic contribution of the dopaminergic system to RL.

There are several limitations of our study. One factor that may have contributed to poor acquisition of contingencies among patients with SZ is worse general neurocognitive performance in comparison with HC participants. However, analyses covarying for RBANS total score indicated that group differences and group x reward contingency interaction remained significant at the trend level. Another potential limitation of the current research is that we were unable to determine the cause of the learning deficit. Learning deficits could be attributed to deficits in learning from PE signaling or deficits in value representation, and the PST task does not allow to isolate these variables during the training phase of the task. A modified version of the task could be used in the future to allow for association of specific genetic polymorphisms with PFC and BG functionality. Moreover, it should be noted that there are several other dopaminergic receptors that should be investigated in the future [99], especially D3 receptors, which has been involved in pathophysiological mechanisms underlying cognitive impairment observed in patients with SZ [100] as well as in RL [101]. Finally, epistatic interactions between dopaminergic genes should be given more attention, since looking at the effects of multiple genes on a single trait provides a comprehensive and more reliable way to determine genetic effects on endophenotype, as shown for example in a study on the combined effect of *COMT* (rs4680) and *DRD3* (rs6289) on cognition in SZ [102] or response to treatment [103]. It should also be mentioned that considering the candidate gene approach, we included relatively small sample of participant. However, previous genetic studies on the RL tasks suggest that the cognitive measures employed in this area of research point to relatively large effects of genetic variations in dopaminergic function [25].

## 5. Conclusions

In conclusion, SZ patients performed significantly worse on the probabilistic RL task in comparison to HC participants. Average performance during the training phase was associated with general neurocognitive functioning, but not with current symptomatology. We found no significant association between dopaminergic genetic polymorphisms and probabilistic RL among SZ patients; however, among HC participants with respect to the *DAT1* rs28363170 polymorphism, individuals with 10-alle repeat genotypes performed significantly better in comparison to 9-allele repeat carriers (9R/9R and 9R/10R genotypes).

## Figures and Tables

**Table 1:** General characteristic of the SZ patients and HC participants.

Variable	SZ	HC	*p*-Value
Demographic information			
Age (years)	37.61 ± 12.99	39.07 ± 18.74	0.474
Gender (M/F)	58/52	66/122	0.003
Educational level (%) Primary Vocational Secondary Higher	19.8% 9.9% 44% 26.4%	11.7% 0.0% 48.7% 39.6%	0.003
Neurocognition (mean ± SD)			
RBANS—immediate memory	39.03 ± 10.86	50.12 ± 6.87	<0.001
RBANS—visuospatial/constructional	33.39 ± 6.44	37.09 ± 3.13	<0.001
RBANS—language	29.19 ± 6.37	33.96 ± 6.38	<0.001
RBANS—attention	44.76 ± 13.84	61.49 ± 15.20	<0.001
RBANS—delayed memory	42.64 ± 11.47	53.70 ± 6.09	<0.001
RBANS—total score	189.02 ± 40.32	236.08 ± 29.96	<0.001
Clinical ratings (mean ± SD)			
Age of onset	24.82 ± 7.38	-	-
Illness duration	11.12 ± 10.38	-	-
BPRS	41.73 ± 9.64	-	-
PANSS positive symptoms	13.64 ± 4.63	-	-
PANSS negative symptoms	21.07 ± 9.28	-	-
PANSS general symptoms	30.10 ± 7.76	-	-
SANS	22.57 ± 19.81	-	-
SAPS	35.43 ± 21.78	-	-
MADRS	8.19 ± 8.69	-	-
GAF	46.08 ± 17.77	-	-
Antipsychotic medication (mean ± SD)		-	-
CPZ	482.12 ± 306.99	-	-

Abbreviations: RBANS—Repeatable Battery for the Assessment of Neuropsychological Status; BPRS—Brief Psychiatric Rating Scale; PANSS—Positive and Negative Syndrome Scale; SANS—Scale for the Assessment of Negative Symptoms; SAPS—Scale for the Assessment of Positive Symptoms; MADRS—Montgomery–Asberg Depression Rating Scale; GAF—Global Assessment of Functioning scale; SZ—schizophrenia-spectrum patients; HC—healthy control participants.

**Table 2:** Acquisition of probabilistic contingencies for AB, CD, and EF conditions.

Models	Variable	F	*p*-Value
Model 1 (*R*^2^ = 0.60, *p*-value < 0.001)	Group	15.52	<0.001
	Reward contingency	1.13	0.324
	Group x reward contingency	3.07	0.047
Model 2 (*R*^2^ = 0.064, *p*-value < 0.001)	Group	13.79	<0.001
	Reward contingency	1.13	0.323
	Group x reward contingency	2.77	0.064
	Gender	1.83	0.176
Model 3 (*R*^2^ = 0.066, *p*-value < 0.001)	Group	4.43	0.032
	Reward contingency	1.51	0.223
	Group x reward contingency	2.77	0.064
	Educational level	4.43	0.036
Model 4 (*R*^2^ = 0.072, *p*-value < 0.001)	Group	1.70	0.193
	Reward contingency	1.53	0.217
	Group x reward contingency	2.77	0.064
	RBANS total score	4.58	0.033
Model 5 (*R*^2^ = 0.073, *p*-value = 0.001)	Group	0.72	0.397
	Reward contingency	1.61	0.202
	Group x reward contingency	2.91	0.056
	Gender	0.88	0.350
	Educational level	1.48	0.224
	RBANS total score	1.75	0.187

Abbreviations: RBANS—Repeatable Battery for the Assessment of Neuropsychological Status, group: healthy control (HC), schizophrenia (SZ), reward contingency: AB (80%/20%), CD (70%, 30%), EF (80%, 20%).

**Table 3:** Association between genetic polymorphisms and acquisition and test performance on PST in the whole group and with respect to SZ patients and HC participants.

Genetic Polymorphism	Task Phase	Variable	*p*-Value		
			SZ + HC	SZ	HC
*DRD2* rs2734839	Training phase	Accuracy in all trials	0.482	0.439	0.915
	Test phase	Choose-A frequency	0.445	0.357	0.882
		Avoid-B frequency	0.271	0.397	0.645
*DRD2* rs936461	Training phase	Accuracy in all trials	0.505	0.424	0.462
	Test phase	Choose-A frequency	0.055	0.093	0.575
		Avoid-B frequency	0.569	0.385	0.990
*DRD2* rs1800497	Training phase	Accuracy in all trials	0.089	0.159	0.373
	Test phase	Choose-A frequency	0.784	0.513	0.742
		Avoid-B frequency	0.608	0.484	0.254
*DRD2* rs1799732	Training phase	Accuracy in all trials	0.480	0.442	0.116
	Test phase	Choose-A frequency	0.821	0.657	0.088
		Avoid-B frequency	0.951	0.989	0.842
*DRD2* rs6277	Training phase	Accuracy in all trials	0.379	0.822	0.652
	Test phase	Choose-A frequency	0.529	0.180	0.113
		Avoid-B frequency	0.936	0.871	0.161
*DRD4* rs1800955	Training phase	Accuracy in all trials	0.531	0.814	0.380
	Test phase	Choose-A frequency	**0.039**	0.300	**0.047**
		Avoid-B frequency	0.543	0.338	0.842
*DRD4* rs747302	Training phase	Accuracy in all trials	0.656	*0.402*	0.784
	Test phase	Choose-A frequency	0.469	*0.450*	0.958
		Avoid-B frequency	0.548	*0.699*	0.592
*COMT* rs4680	Training phase	Accuracy in all trials	0.111	0.673	**0.035**
	Test phase	Choose-A frequency	0.803	0.697	0.577
		Avoid-B frequency	0.641	0.139	0.836
*DAT1* rs2975226	Training phase	Accuracy in all trials	0.294	**0.018**	0.746
	Test phase	Choose-A frequency	0.408	0.501	*0.275*
		Avoid-B frequency	0.154	0.281	*0.305*
*DAT1* rs28363170	Training phase	Accuracy in all trials	**0.042**	0.469	**0.004**
	Test phase	Choose-A frequency	0.702	0.528	0.897
		Avoid-B frequency	0.436	0.495	0.593
*DARP32* rs907094	Training phase	Accuracy in all trials	0.665	0.469	0.747
	Test phase	Choose-A frequency	0.870	0.528	0.897
		Avoid-B frequency	0.598	0.495	0.646

Abbreviations: group: healthy control (HC), schizophrenia (SZ); *DRD2*—gene encoding dopaminergic D2 receptor, *DRD4*—gene encoding dopaminergic D4 receptor, *COMT*—gene encoding catechol-o-methyltransferase, *DAT1*—gene encoding dopamine transporter gene, *DARP32*—gene encoding dopamine and cAMP-regulated phosphoprotein of molecular weight 32 kDa, significant associations (*p*-value less than 0.05) are marked in bold, distributions that did not follow the HWE are marked in italic.

## Data Availability

The data presented in this study is available on request from the corresponding author.

## Appendix A

**Table A1:** Distribution of genotypes in SZ patients and HC participants.

Genetic Polymorphism	Genotype	SZ	HC	*p*-Value
*DRD2* rs2734839	CC	23 (29.9%)	53 (21.4%)	0.229
	CT	35 (45.5%)	97 (44.7%)	
	TT	19 (24.7%)	67 (34.3%)	
*DRD2* rs936461	AA	13 (16.9%)	18 (12.9%)	0.157
	AG	37 (48.1%)	54 (38.6%)	
	GG	27 (35.1%)	68 (48.6%)	
*DRD2* rs1800497	CC	49 (63.6%)	97 (69.8%)	0.396
	CT	24 (31.2%)	39 (28.1%)	
	TT	4 (5.2%)	3 (2.2%)	
*DRD2* rs1799732	GG	61 (80.3%)	118 (86.1%)	0.516
	G-	13 (17.1%)	17 (12.4%)	
	--	2 (2.6%)	2 (1.5%)	
*DRD2* rs6277	AA	16 (20.8%)	61 (32.1%)	0.059
	AG	35 (45.5%)	101 (47.1%)	
	GG	26 (33.8%)	55 (20.7%)	
*DRD4* rs1800955	CC	20 (26.3%)	37 (26.4%)	0.430
	CT	39 (51.3%)	61 (43.6%)	
	TT	17 (22.4%)	42 (30.0%)	
*DRD4* rs747302	CC	7 (13.5%)	18 (24.7%)	0.081
	CG	9 (17.3%)	19 (26.0%)	
	GG	36 (69.2%)	36 (49.3%)	
*COMT* rs4680	Met/Met	27 (35.1%)	31 (22.1%)	0.101
	Met/Val	35 (45.5%)	71 (50.7%)	
	Val/Val	15 (19.5%)	38 (27.1%)	
*DAT1* rs2975226	AA	20 (26.7%)	38 (27.5%)	0.978
	AT	31 (41.3%)	55 (39.9%)	
	TT	24 (32.0%)	45 (32.6%)	
*DAT1* rs28363170	9R, 9R	3 (4.0%)	10 (7.3%)	0.583
	9R, 10R	31 (41.3%)	51 (37.2%)	
	10R, 10R	41 (54.7%)	76 (55.5%)	
*DARP32* rs907094	AA	42 (55.3%)	61 (43.6%)	0.172
	AG	30 (39.5%)	64 (45.7%)	
	GG	4 (5.3%)	15 (10.7%)	

Abbreviations: group: healthy control (HC), schizophrenia (SZ); *DRD2*—gene encoding dopaminergic D2 receptor, *DRD4*—gene encoding dopaminergic D4 receptor, *COMT*—gene encoding catechol-o-methyltransferase, *DAT1*—gene encoding dopamine transporter gene, *DARP32*—gene encoding dopamine and cAMP-regulated phosphoprotein of molecular weight 32 kDa, significant associations (*p*-value less than 0.05) are marked in bold, distributions that did not follow the HWE are marked in italic, 9R and 10R—nine- and ten-repeat alleles, the number of genotypes for each studied polymorphism differ due to poor DNA quality and/or unsuccessful genotyping.

**Table A2:** Correlational analysis between training and test phase performance on PST with neurocognitive and clinical ratings.

Variable	Learning Accuracy in Training Phase	Choose-A Frequency in Test Phase	Avoid-B Frequency in Test Phase
Neurocognition			
RBANS—immediate memory	*r* ** = 0.30, *p* < 0.001	*r* ** = −0.23, *p* = 0.791	*r* ** = −0.12, *p* = 0.154
RBANS—visuospatial constructional	*r* ** = 0.23, *p* = 0.007	*r* ** = 0.15, *p* = 0.846	*r* ** = −0.11, *p* = 0.202
RBANS—language	*r* * = 0.15, *p* = 0.082	*r* * = −0.10, *p* = 0.232	*r* * = −0.58, *p* = 0.500
RBANS—attention	*r* * = 0.26, *p* = 0.002	*r* * = −0.70, *p* = 0.446	*r* * = −0.12, *p* = 0.156
RBANS—delayed memory	*r* ** = 0.30, *p* < 0.001	*r* ** = −0.4, *p* = 0.629	*r* ** = −0.12, *p* = 0.176
RBANS—total score	*r* * = 0.30, *p* < 0.001	*r* * = −0.65, *p* = 0.448	*r* * = −0.12, *p* = 0.154
Clinical ratings			
Age of onset	*r* * = −0.02, *p* = 0.200	*r* * = −0.15, *p* = 0.341	*r* * = 0.11, *p* = 0.510
Illness duration	*r* ** = −0.08, *p* = 0.337	*r* ** = 0.03, *p* = 0.831	*r* ** = 0.12, *p* = 0.465
BPRS	*r* * = −0.13, *p* = 0.418	*r* * = −0.45, *p* = 0.788	*r* * = 0.32, *p* = 0.050
PANSS—positive symptoms	*r* * = −0.25, *p* = 0.418	*r* * = −0.47, *p* = 0.757	*r* * = 0.17, *p* = 0.259
PANSS—negative symptoms	*r* * = 0.30, *p* = 0.845	*r* * = 0.76, *p* = 0.618	*r* * = 0.18, *p* = 0.258
PANSS—general symptoms	*r* * = −0.2, *p* = 0.436	*r* * = −0.52, *p* = 0.736	*r* * = 0.01, *p* = 0.258
SANS	*r* * = −0.07, *p* = 0.709	*r* * = −0.18, *p* = 0.921	*r* * = 0.25, *p* = 0.157
SAPS	*r* ** < 0.01, *p* > 0.99	*r* ** = −0.17, *p* = 0.389	*r* ** = 0.14, *p* = 0.469
MADRS	*r* ** = −0.16, *p* = 0.395	*r* ** = 0.15, *p* = 0.422	*r* ** = 0.2, *p* = 0.907
GAF	*r* * = −0.17, *p* = 0.33	*r* * = −0.21, *p* = 0.240	*r* * = 0.11, *p* = 0.530
Antipsychotic medication			
CPZ	*r* ** = −0.16, *p* = 0.330	*r* ** = 0.43, *p* = 0.793	*r* ** = 0.12, *p* = 0.458

Abbreviations: RBANS—Repeatable Battery for the Assessment of Neuropsychological Status, BPRS—Brief Psychiatric Rating Scale, PANSS—Positive and Negative Syndrome Scale, SANS—Scale for the Assessment of Negative Symptoms, SAPS—Scale for the Assessment of Positive Symptoms, MDRS—Montgomery–Asberg Depression Rating Scale, GAF—Global Assessment of Functioning scale, * Pearson’s correlation coefficient, ** Spearman’s correlation coefficient.

**Table A3:** Correlational analysis between neurocognitive functioning and clinical ratings.

Variable	SZ
Age of onset	*r* * = 0.08, *p* = 0.427
Illness duration	*r* ** = −0.10, *p* = 0.422
BPRS	*r* * = −0.26, *p* = 0.021
PANSS P—positive symptoms	*r* * = −0.15, *p* = 0.132
PANSS N—negative symptoms	*r* * = −0.34, *p* = 0.001
PANSS G—general symptoms	*r* * = −0.21, *p* = 0.0.49
SANS	*r* * = −0.14, *p* = 0.212
SAPS	*r* ** = −0.10, *p* = 0.374
MADRS	*r* ** = −0.10, *p* = 0.422
GAF	*r* * = 0.29, *p* = 0.011
CPZ	*r* ** = −0.23, *p* = 0.027

Abbreviations: BPRS—Brief Psychiatric Rating Scale, PANSS—Positive and Negative Syndrome Scale, SANS—Scale for the Assessment of Negative Symptoms, SAPS—Scale for the Assessment of Positive Symptoms, MDRS—Montgomery–Asberg Depression Rating Scale, GAF—Global Assessment of Functioning scale, * Pearson’s correlation coefficient, ** Spearman’s correlation coefficient.

## References

[1] Frydecka D., Eissa A.M., Hewedi D.H., Ali M., Drapala J., Misiak B., Klosinska E., Phillips J.R., Moustafa A.A. (2014). Impairments of working memory in schizophrenia and bipolar disorder: The effect of history of psychotic symptoms and different aspects of cognitive task demands. Front. Behav. Neurosci..

[2] Howes O.D., Murray R.M. (2014). Schizophrenia: An integrated sociodevelopmental-cognitive model. Lancet.

[3] Frydecka D., Misiak B., Pawlak-Adamska E., Karabon L., Tomkiewicz A., Sedlaczek P., Kiejna A., Beszlej J.A. (2015). Interleukin-6: The missing element of the neurocognitive deterioration in schizophrenia? The focus on genetic underpinnings, cognitive impairment and clinical manifestation. Eur. Arch. Psychiatry Clin. Neurosci..

[4] Howes O.D., Kambeitz J., Kim E., Stahl D., Slifstein M., Abi-Dargham A., Kapur S. (2012). The nature of dopamine dysfunction in schizophrenia and what this means for treatment. Arch. Gen. Psychiatry.

[5] Howes O.D., Kapur S. (2009). The dopamine hypothesis of schizophrenia: Version III—The final common pathway. Schizophr. Bull..

[6] Kapur S. (2003). Psychosis as a state of aberrant salience: A framework linking biology, phenomenology, and pharmacology in schizophrenia. Am. J. Psychiatry.

[7] Houk J.C. (2007). Biological implementation of the temporal difference algorithm for reinforcement learning: Theoretical comment on O’Reilly et al. (2007). Behav. Neurosci..

[8] Schultz W. (2001). Reward signaling by dopamine neurons. Neuroscientist.

[9] Schultz W. (2002). Getting formal with dopamine and reward. Neuron.

[10] Frank M.J. (2008). Schizophrenia: A computational reinforcement learning perspective. Schizophr. Bull..

[11] Smith A., Li M., Becker S., Kapur S. (2006). Dopamine, prediction error and associative learning: A model-based account. Network.

[12] Millard S.J., Bearden C.E., Karlsgodt K.H., Sharpe M.J. (2021). The prediction-error hypothesis of schizophrenia: New data point to circuit-specific changes in dopamine activity. Neuropsychopharmacology.

[13] Murray G.K., Corlett P.R., Clark L., Pessiglione M., Blackwell A.D., Honey G., Jones P.B., Bullmore E.T., Robbins T.W., Fletcher P.C. (2008). Substantia nigra/ventral tegmental reward prediction error disruption in psychosis. Mol. Psychiatry.

[14] Corlett P.R., Murray G.K., Honey G.D., Aitken M.R., Shanks D.R., Robbins T.W., Bullmore E.T., Dickinson A., Fletcher P.C. (2007). Disrupted prediction-error signal in psychosis: Evidence for an associative account of delusions. Brain.

[15] Waltz J.A., Gold J.M. (2007). Probabilistic reversal learning impairments in schizophrenia: Further evidence of orbitofrontal dysfunction. Schizophr. Res..

[16] Murray G.K., Clark L., Corlett P.R., Blackwell A.D., Cools R., Jones P.B., Robbins T.W., Poustka L. (2008). Incentive motivation in first-episode psychosis: A behavioural study. BMC Psychiatry.

[17] Gold J.M., Waltz J.A., Matveeva T.M., Kasanova Z., Strauss G.P., Herbener E.S., Collins A.G., Frank M.J. (2012). Negative symptoms and the failure to represent the expected reward value of actions: Behavioral and computational modeling evidence. Arch. Gen. Psychiatry.

[18] Geana A., Barch D.M., Gold J.M., Carter C.S., MacDonald A.W., Ragland J.D., Silverstein S.M., Frank M.J. (2021). Using Computational Modeling to Capture Schizophrenia-Specific Reinforcement Learning Differences and Their Implications on Patient Classification. Biol. Psychiatry Cogn. Neurosci. Neuroimaging.

[19] Edwards A.C., Bacanu S.A., Bigdeli T.B., Moscati A., Kendler K.S. (2016). Evaluating the dopamine hypothesis of schizophrenia in a large-scale genome-wide association study. Schizophr. Res..

[20] Howes O.D., McCutcheon R., Owen M.J., Murray R.M. (2017). The Role of Genes, Stress, and Dopamine in the Development of Schizophrenia. Biol. Psychiatry.

[21] Kasanova Z., Ceccarini J., Frank M.J., van Amelsvoort T., Booij J., van Duin E., Steinhart H., Vaessen T., Heinzel A., Mottaghy F. (2018). Intact striatal dopaminergic modulation of reward learning and daily-life reward-oriented behavior in first-degree relatives of individuals with psychotic disorder. Psychol. Med..

[22] Ermakova A.O., Knolle F., Justicia A., Bullmore E.T., Jones P.B., Robbins T.W., Fletcher P.C., Murray G.K. (2018). Abnormal reward prediction-error signalling in antipsychotic naive individuals with first-episode psychosis or clinical risk for psychosis. Neuropsychopharmacology.

[23] Montagnese M., Knolle F., Haarsma J., Griffin J.D., Richards A., Vertes P.E., Kiddle B., Fletcher P.C., Jones P.B., Owen M.J. (2020). Reinforcement learning as an intermediate phenotype in psychosis? Deficits sensitive to illness stage but not associated with polygenic risk of schizophrenia in the general population. Schizophr. Res..

[24] Lancaster T.M., Linden D.E., Tansey K.E., Banaschewski T., Bokde A.L., Bromberg U., Buchel C., Cattrell A., Conrod P.J., Flor H. (2016). Polygenic Risk of Psychosis and Ventral Striatal Activation During Reward Processing in Healthy Adolescents. JAMA Psychiatry.

[25] Frank M.J., Moustafa A.A., Haughey H.M., Curran T., Hutchison K.E. (2007). Genetic triple dissociation reveals multiple roles for dopamine in reinforcement learning. Proc. Natl. Acad. Sci. USA.

[26] Jocham G., Klein T.A., Neumann J., von Cramon D.Y., Reuter M., Ullsperger M. (2009). Dopamine DRD2 polymorphism alters reversal learning and associated Neural. activity. J. Neurosci..

[27] Doll B.B., Hutchison K.E., Frank M.J. (2011). Dopaminergic genes predict individual differences in susceptibility to confirmation bias. J. Neurosci..

[28] Krugel L.K., Biele G., Mohr P.N., Li S.C., Heekeren H.R. (2009). Genetic variation in dopaminergic neuromodulation influences the ability to rapidly and flexibly adapt decisions. Proc. Natl. Acad. Sci. USA.

[29] Frank M.J., Hutchison K. (2009). Genetic contributions to avoidance-based decisions: Striatal D2 receptor polymorphisms. Neuroscience.

[30] Richter A., de Boer L., Guitart-Masip M., Behnisch G., Seidenbecher C.I., Schott B.H. (2021). Motivational learning biases are differentially modulated by genetic determinants of striatal and prefrontal dopamine function. J. Neural. Transm..

[31] Corral-Frias N.S., Pizzagalli D.A., Carre J.M., Michalski L.J., Nikolova Y.S., Perlis R.H., Fagerness J., Lee M.R., Conley E.D., Lancaster T.M. (2016). COMT Val(158) Met genotype is associated with reward learning: A replication study and meta-analysis. Genes Brain Behav..

[32] Tardiff N., Graves K.N., Thompson-Schill S.L. (2018). The Role of Frontostriatal Systems in Instructed Reinforcement Learning: Evidence From Genetic and Experimentally-Induced Variation. Front. Hum. Neurosci..

[33] McGuffin P., Farmer A., Harvey I. (1991). A polydiagnostic application of operational criteria in studies of psychotic illness. Development and reliability of the OPCRIT system. Arch. Gen. Psychiatry.

[34] Andreasen N.C., Olsen S. (1982). Negative v positive schizophrenia. Definition and validation. Arch. Gen. Psychiatry.

[35] Kay S.R., Fiszbein A., Opler L.A. (1987). The positive and negative syndrome scale (PANSS) for schizophrenia. Schizophr. Bull..

[36] Montgomery S.A., Asberg M. (1979). A new depression scale designed to be sensitive to change. Br. J. Psychiatry.

[37] Beck A.T., Ward C.H., Mendelson M., Mock J., Erbaugh J. (1961). An inventory for measuring depression. Arch. Gen. Psychiatry.

[38] Woods S.W. (2003). Chlorpromazine equivalent doses for the newer atypical antipsychotics. J. Clin. Psychiatry.

[39] Randolph C., Tierney M.C., Mohr E., Chase T.N. (1998). The Repeatable Battery for the Assessment of Neuropsychological Status (RBANS): Preliminary clinical validity. J. Clin. Exp. NeuroPsychol..

[40] Frank M.J., Seeberger L.C., O’Reilly C.R. (2004). By carrot or by stick: Cognitive reinforcement learning in parkinsonism. Science.

[41] Waltz J.A., Frank M.J., Robinson B.M., Gold J.M. (2007). Selective reinforcement learning deficits in schizophrenia support predictions from computational models of striatal-cortical dysfunction. Biol. Psychiatry.

[42] Meyer-Lindenberg A., Straub R.E., Lipska B.K., Verchinski B.A., Goldberg T., Callicott J.H., Egan M.F., Huffaker S.S., Mattay V.S., Kolachana B. (2007). Genetic evidence implicating DARPP-32 in human frontostriatal structure, function, and cognition. J. Clin. Investig..

[43] Ritchie T.E., Noble P. (2003). Association of seven polymorphisms of the D2 dopamine receptor gene with brain receptor-binding characteristics. Neurochem. Res..

[44] Eisenstein S.A., Bogdan R., Love-Gregory L., Corral-Frias N.S., Koller J.M., Black K.J., Moerlein S.M., Perlmutter J.S., Barch D.M., Hershey T. (2016). Prediction of striatal D2 receptor binding by DRD2/ANKK1 TaqIA allele status. Synapse.

[45] Voisey J., Swagell C.D., Hughes I.P., Lawford B.R., Young R.M., Morris C.P. (2012). A novel DRD2 single-nucleotide polymorphism associated with schizophrenia predicts age of onset: HapMap tag-single-nucleotide polymorphism analysis. Genet. Test. Mol. Biomark..

[46] Duan J., Wainwright M.S., Comeron J.M., Saitou N., Sanders A.R., Gelernter J., Gejman P.V. (2003). Synonymous mutations in the human dopamine receptor D2 (DRD2) affect mRNA stability and synthesis of the receptor. Hum. Mol. Genet..

[47] Hirvonen M., Laakso A., Nagren K., Rinne J.O., Pohjalainen T., Hietala J. (2004). C957T polymorphism of the dopamine D2 receptor (DRD2) gene affects striatal DRD2 availability in vivo. Mol. Psychiatry.

[48] Laakso A., Pohjalainen T., Bergman J., Kajander J., Haaparanta M., Solin O., Syvalahti E., Hietala J. (2005). The A1 allele of the human D2 dopamine receptor gene is associated with increased activity of striatal L-amino acid decarboxylase in healthy subjects. Pharmacogenet. Genom..

[49] Jonsson E.G., Nothen M.M., Grunhage F., Farde L., Nakashima Y., Propping P., Sedvall G.C. (1999). Polymorphisms in the dopamine D2 receptor gene and their relationships to striatal dopamine receptor density of healthy volunteers. Mol. Psychiatry.

[50] Forbes E.E., Brown S.M., Kimak M., Ferrell R.E., Manuck S.B., Hariri A.R. (2009). Genetic variation in components of dopamine neurotransmission impacts ventral striatal reactivity associated with impulsivity. Mol. Psychiatry.

[51] Okuyama Y., Ishiguro H., Toru M., Arinami T. (1999). A genetic polymorphism in the promoter region of DRD4 associated with expression and schizophrenia. Biochem. Biophys. Res. Commun..

[52] Kramer U.M., Cunillera T., Camara E., Marco-Pallares J., Cucurell D., Nager W., Bauer P., Schule R., Schols L., Rodriguez-Fornells A. (2007). The impact of catechol-O-methyltransferase and dopamine D4 receptor genotypes on neurophysiological markers of performance monitoring. J. Neurosci..

[53] Lowe N., Kirley A., Mullins C., Fitzgerald M., Gill M., Hawi Z. (2004). Multiple marker analysis at the promoter region of the DRD4 gene and ADHD: Evidence of linkage and association with the SNP -616. Am. J. Med. Genet. B Neuropsychiatr. Genet..

[54] Huang S.Y., Chen H.K., Ma K.H., Shy M.J., Chen J.H., Lin W.C., Lu R.B. (2010). Association of promoter variants of human dopamine transporter gene with schizophrenia in Han Chinese. Schizophr. Res..

[55] Fuke S., Suo S., Takahashi N., Koike H., Sasagawa N., Ishiura S. (2001). The VNTR polymorphism of the human dopamine transporter (DAT1) gene affects gene expression. Pharm. J..

[56] VanNess S.H., Owens M.J., Kilts C.D. (2005). The variable number of tandem repeats element in DAT1 regulates in vitro dopamine transporter density. BMC Genet..

[57] Garcia-Garcia M., Barcelo F., Clemente I.C., Escera C. (2010). The role of the dopamine transporter DAT1 genotype on the Neural. correlates of cognitive flexibility. Eur. J. Neurosci..

[58] Stollstorff M., Foss-Feig J., Cook E.H.J., Stein M.A., Gaillard W.D., Vaidya C.J. (2010). Neural response to working memory load varies by dopamine transporter genotype in children. Neuroimage.

[59] Simon J.R., Stollstorff M., Westbay L.C., Vaidya C.J., Howard J., Howard D.V. (2011). Dopamine transporter genotype predicts implicit sequence learning. Behav. Brain Res..

[60] Weickert T.W., Mattay V.S., Das S., Bigelow L.B., Apud J.A., Egan M.F., Weinberger D.R., Goldberg T.E. (2013). Dopaminergic therapy removal differentially effects learning in schizophrenia and Parkinson’s disease. Schizophr. Res..

[61] Keri S., Juhasz A., Rimanoczy A., Szekeres G., Kelemen O., Cimmer C., Szendi I., Benedek G., Janka Z. (2005). Habit learning and the genetics of the dopamine D3 receptor: Evidence from patients with schizophrenia and healthy controls. Behav. Neurosci..

[62] Horan W.P., Green M.F., Knowlton B.J., Wynn J.K., Mintz J., Nuechterlein K.H. (2008). Impaired implicit learning in schizophrenia. Neuropsychology.

[63] Weickert T.W., Goldberg T.E., Callicott J.H., Chen Q., Apud J.A., Das S., Zoltick B.J., Egan M.F., Meeter M., Myers C. (2009). Neural. correlates of probabilistic category learning in patients with schizophrenia. J. Neurosci..

[64] Weickert T.W., Terrazas A., Bigelow L.B., Malley J.D., Hyde T., Egan M.F., Weinberger D.R., Goldberg T.E. (2002). Habit and skill learning in schizophrenia: Evidence of normal striatal processing with abnormal cortical input. Learn. Mem..

[65] Moustafa A.A., Keri S., Somlai Z., Balsdon T., Frydecka D., Misiak B., White C. (2015). Drift diffusion model of reward and punishment learning in schizophrenia: Modeling and experimental data. Behav. Brain Res..

[66] Somlai Z., Moustafa A.A., Keri S., Gluck C.E.M.A. (2011). General functioning predicts reward and punishment learning in schizophrenia. Schizophr. Res..

[67] Strauss G.P., Thaler N.S., Matveeva T.M., Vogel S.J., Sutton G.P., Lee B.G., Allen D.N. (2015). Predicting psychosis across diagnostic boundaries: Behavioral and computational modeling evidence for impaired reinforcement learning in schizophrenia and bipolar disorder with a history of psychosis. J. Abnorm. Psychol..

[68] Cicero D.C., Martin E.A., Becker T.M., Kerns J.G. (2014). Reinforcement learning deficits in people with schizophrenia persist after extended trials. Psychiatry Res..

[69] Waltz J.A., Frank M.J., Wiecki T.V., Gold J.M. (2011). Altered probabilistic learning and response biases in schizophrenia: Behavioral evidence and neurocomputational modeling. Neuropsychology.

[70] Deserno L., Boehme R., Heinz A., Schlagenhauf F. (2013). Reinforcement learning and dopamine in schizophrenia: Dimensions of symptoms or specific features of a disease group?. Front. Psychiatry.

[71] Strauss G.P., Frank M.J., Waltz J.A., Kasanova Z., Herbener E.S., Gold J.M. (2011). Deficits in positive reinforcement learning and uncertainty-driven exploration are associated with distinct aspects of negative symptoms in schizophrenia. Biol. Psychiatry.

[72] Shen W., Flajolet M., Greengard P., Surmeier D.J. (2008). Dichotomous dopaminergic control of striatal synaptic plasticity. Science.

[73] Kapur S., Seeman P. (2001). Does fast dissociation from the dopamine d(2) receptor explain the action of atypical antipsychotics? A new hypothesis. Am. J. Psychiatry.

[74] Nord M., Farde L. (2011). Antipsychotic occupancy of dopamine receptors in schizophrenia. CNS Neurosci. Ther..

[75] Frydecka D., Beszlej J.A., Goscimski P., Kiejna A., Misiak B. (2016). Profiling cognitive impairment in treatment-resistant schizophrenia patients. Psychiatry Res..

[76] Torrisi S.A., Laudani S., Contarini G., de Luca A., Geraci F., Manago F., Papaleo F., Salomone S., Drago F., Leggio G.M. (2020). Dopamine, Cognitive Impairments and Second-Generation Antipsychotics: From Mechanistic Advances to More Personalized Treatments. Pharmaceuticals.

[77] Gold J.M., Strauss G.P., Waltz J.A., Robinson B.M., Brown J.K., Frank M.J. (2013). Negative symptoms of schizophrenia are associated with abnormal effort-cost computations. Biol. Psychiatry.

[78] Strauss G.P., Datta R., Armstrong W., Raugh I.M., Kraguljac N.V., Lahti A.C. (2021). Reinforcement learning abnormalities in the attenuated psychosis syndrome and first episode psychosis. Eur. Neuropsychopharmacol..

[79] Klein T.A., Neumann J., Reuter M., Hennig J., von Cramon D.Y., Ullsperger M. (2007). Genetically determined differences in learning from errors. Science.

[80] Cohen M.X., Young J., Baek J.M., Kessler C., Ranganath C. (2005). Individual differences in extraversion and dopamine genetics predict Neural. reward responses. Brain Res. Cogn. Brain Res..

[81] Friedman N.P., Miyake A., Young S.E., Defries J.C., Corley R.P., Hewitt J.K. (2008). Individual differences in executive functions are almost entirely genetic in origin. J. Exp. Psychol. Gen..

[82] Vandenbergh D.J., Persico A.M., Hawkins A.L., Griffin C.A., Li X., Jabs E.W., Uhl G.R. (1992). Human dopamine transporter gene (DAT1) maps to chromosome 5p15.3 and displays a VNTR. Genomics.

[83] Mill J., Asherson P., Browes C., Craig U.D. (2002). Expression of the dopamine transporter gene is regulated by the 3′ UTR VNTR: Evidence from brain and lymphocytes using quantitative RT-PCR. Am. J. Med. Genet..

[84] Faraone S.V., Spencer T.J., Madras B.K., Zhang-James Y., Biederman J. (2014). Functional effects of dopamine transporter gene genotypes on in vivo dopamine transporter functioning: A meta-analysis. Mol. Psychiatry.

[85] Lewis D.A., Melchitzky D.S., Sesack S.R., Whitehead R.E., Auh S., Sampson A. (2001). Dopamine transporter immunoreactivity in monkey cerebral cortex: Regional, laminar, and ultrastructural localization. J. Comp. Neurol..

[86] Bertolino A., Fazio L., di Giorgio A., Blasi G., Romano R., Taurisano P., Caforio G., Sinibaldi L., Ursini G., Popolizio T. (2009). Genetically determined interaction between the dopamine transporter and the D2 receptor on prefronto-striatal activity and volume in humans. J. Neurosci..

[87] Schuck N.W., Frensch P.A., Schjeide B.M., Schroder J., Bertram L., Li S.C. (2013). Effects of aging and dopamine genotypes on the emergence of explicit memory during sequence learning. Neuropsychologia.

[88] Althaus M., Groen Y., Wijers A.A., Minderaa R.B., Kema I.P., Dijck J.D., Hoekstra P.J. (2010). Variants of the SLC6A3 (DAT1) polymorphism affect performance monitoring-related cortical evoked potentials that are associated with ADHD. Biol. Psychol..

[89] Biehl S.C., Dresler T., Reif A., Scheuerpflug P., Deckert J., Herrmann M.J. (2011). Dopamine transporter (DAT1) and dopamine receptor D4 (DRD4) genotypes differentially impact on electrophysiological correlates of error processing. PLoS ONE.

[90] Raczka K.A., Mechias M.L., Gartmann N., Reif A., Deckert J., Pessiglione M., Kalisch R. (2011). Empirical support for an involvement of the mesostriatal dopamine system in human fear extinction. Transl. Psychiatry.

[91] Dubol M., Trichard C., Leroy C., Sandu A.L., Rahim M., Granger B., Tzavara E.T., Karila L., Martinot J.L., Artiges E. (2018). Dopamine Transporter and Reward Anticipation in a Dimensional Perspective: A Multimodal Brain Imaging Study. Neuropsychopharmacology.

[92] Dreher J.C., Kohn P., Kolachana B., Weinberger D.R., Berman K.F. (2009). Variation in dopamine genes influences responsivity of the human reward system. Proc. Natl. Acad. Sci. USA.

[93] Aarts E., Roelofs A., Franke B., Rijpkema M., Fernandez G., Helmich R.C., Cools R. (2010). Striatal dopamine mediates the interface between motivational and cognitive control in humans: Evidence from genetic imaging. Neuropsychopharmacology.

[94] Yacubian J., Sommer T., Schroeder K., Glascher J., Kalisch R., Leuenberger B., Buchel D.F.B. (2007). Gene-gene interaction associated with Neural. reward sensitivity. Proc. Natl. Acad. Sci. USA.

[95] Den Ouden H.E., Daw N.D., Fernandez G., Elshout J.A., Rijpkema M., Hoogman M., Franke B., Cools R. (2013). Dissociable effects of dopamine and serotonin on reversal learning. Neuron.

[96] Beeler J.A., Daw N., Frazier C.R., Zhuang X. (2010). Tonic dopamine modulates exploitation of reward learning. Front. Behav. Neurosci..

[97] Salamone J.D., Correa M., Farrar A., Mingote S.M. (2007). Effort-related functions of nucleus accumbens dopamine and associated forebrain circuits. Psychopharmacology.

[98] Dreyer J.K., Herrik K.F., Berg R.W., Hounsgaard J.D. (2010). Influence of phasic and tonic dopamine release on receptor activation. J. Neurosci..

[99] Martel J.C., McArthur S.G. (2020). Dopamine Receptor Subtypes, Physiology and Pharmacology: New Ligands and Concepts in Schizophrenia. Front. Pharmacol..

[100] Leggio G.M., Torrisi S.A., Mastrogiacomo R., Mauro D., Chisari M., Devroye C., Scheggia D., Nigro M., Geraci F., Pintori N. (2021). The epistatic interaction between the dopamine D3 receptor and dysbindin-1 modulates higher-order cognitive functions in mice and humans. Mol. Psychiatry.

[101] Groman S.M., Smith N.J., Petrullli J.R., Massi B., Chen L., Ropchan J., Huang Y., Lee D., Morris E.D., Taylor J.R. (2016). Dopamine D3 Receptor Availability Is Associated with Inflexible Decision Making. J. Neurosci..

[102] Loch A.A., van de Bilt M.T., Bio D.S., Prado C.M., de Sousa R.T., Valiengo L.L., Moreno R.A., Zanetti M.V., Gattaz W.F. (2015). Epistasis between COMT Val158Met and DRD3 Ser9Gly polymorphisms and cognitive function in schizophrenia: Genetic influence on dopamine transmission. Braz. J. Psychiatry.

[103] Escamilla R., Camarena B., Saracco-Alvarez R., Fresan A., Hernandez S., Aguilar-Garcia A. (2018). Association study between COMT, DRD2, and DRD3 gene variants and antipsychotic treatment response in Mexican patients with schizophrenia. Neuropsychiatr. Dis. Treat..

